# Health Gain by Salt Reduction in Europe: A Modelling Study

**DOI:** 10.1371/journal.pone.0118873

**Published:** 2015-03-31

**Authors:** Marieke A. H. Hendriksen, Joop M. A. van Raaij, Johanna M. Geleijnse, Joao Breda, Hendriek C. Boshuizen

**Affiliations:** 1 Centre for Nutrition, Prevention and Health Services, National Institute for Public Health and the Environment, Bilthoven, the Netherlands; 2 Division of Human Nutrition, Wageningen University and Research Centre, Wageningen, the Netherlands; 3 Noncommunicable Diseases and Health Promotion, World Health Organization Regional Office for Europe, Copenhagen, Denmark; Geisel School of Medicine at Dartmouth College, UNITED STATES

## Abstract

Excessive salt intake is associated with hypertension and cardiovascular diseases. Salt intake exceeds the World Health Organization population nutrition goal of 5 grams per day in the European region. We assessed the health impact of salt reduction in nine European countries (Finland, France, Ireland, Italy, Netherlands, Poland, Spain, Sweden and United Kingdom). Through literature research we obtained current salt intake and systolic blood pressure levels of the nine countries. The population health modeling tool DYNAMO-HIA including country-specific disease data was used to predict the changes in prevalence of ischemic heart disease and stroke for each country estimating the effect of salt reduction through its effect on blood pressure levels. A 30% salt reduction would reduce the prevalence of stroke by 6.4% in Finland to 13.5% in Poland. Ischemic heart disease would be decreased by 4.1% in Finland to 8.9% in Poland. When salt intake is reduced to the WHO population nutrient goal, it would reduce the prevalence of stroke from 10.1% in Finland to 23.1% in Poland. Ischemic heart disease would decrease by 6.6% in Finland to 15.5% in Poland. The number of postponed deaths would be 102,100 (0.9%) in France, and 191,300 (2.3%) in Poland. A reduction of salt intake to 5 grams per day is expected to substantially reduce the burden of cardiovascular disease and mortality in several European countries.

## Introduction

Hypertension is one of the leading causes of cardiovascular diseases in the European region and contributes substantially to the burden of non-communicable diseases [1]. Prevention of hypertension and thus cardiovascular diseases is important to improve public health.

A major determinant of hypertension is excessive salt intake. There is convincing evidence that reducing salt intake will have a beneficial effect on blood pressure, thereby reducing the incidence of cardiovascular disease [2,3]. A population-wide salt reduction program is considered a cost-effective strategy [4] and to reduce the growing burden of disease related to hypertension and cardiovascular disease, the World health Organization (WHO) recommends reducing salt intake in the general population below 5 grams per day [5].

Currently, salt intake in the WHO European region largely exceeds this population nutrient goal [6]. Therefore, several countries have developed operational salt reduction programs. The most effective salt reduction strategies seem to be those that combine product reformulation, consumer awareness and education, linked to appropriate monitoring mechanisms. Finland and the United Kingdom (UK) have been identified as good examples of successful salt reduction strategies [7]. In those countries salt intake has decreased over the past years [8,9], but in both countries salt intake is still above the population nutrient goal.

Earlier modelling studies concluded that salt reduction would lead to substantial reduction in cardiovascular diseases [10–13]. For example, Barton et al projected a yearly reduction of 4450 cardiovascular deaths when salt intake was reduced by 3 grams per day in England and Wales [10]. Unfortunately, such country projections do not allow for a direct comparison with other countries due to variation in population health modelling tools used and assumptions made.

Hence, we have quantified for nine European countries (Finland, France, Ireland, Italy, the Netherlands, Poland, Spain, Sweden and UK) the health effect of (1) a 30% reduction in current mean population salt intake, according to the WHO global strategy on non-communicable diseases, and the health effect of (2) achieving the WHO population nutrient goal of 5 grams per day using the same population health modelling tool DYNAMO-HIA and the same assumptions. We included countries that were included in the DYNAMO-HIA model, as well as participated in the European Salt Action Network, a network that promotes the harmonization of salt reduction programmes in EU countries. The health effects for ischemic heart disease (IHD) and stroke morbidity and total mortality were examined. Additionally, we examined the robustness of the model in case of missing data and with differences in assumptions made.

## Methods

### Literature study on salt intake and blood pressure levels

We performed an extensive literature search (Scopus) to obtain national representative salt intake (sodium chloride) and blood pressure levels. Government websites of Ministries of Health, National Public Health Institutes or any other government institutes in Europe were additionally searched in order to identify data published in grey literature. We searched for studies in adult populations aged 18 to 90 years that were recently published (after 2000). Preferably data was stratified by sex and age and included a description of the distribution of salt intake and/or blood pressure levels in the population (e.g. by providing both mean and standard deviation (SD)). There were no exclusions based on report language.

Country data that were most recently published were preferred to older publications. In addition, data that was nationally representative was preferred to publications on regional or municipal salt intake or blood pressure. Salt intake based on 24-hour urine collections was preferred to data from dietary surveys as they may not include salt added during cooking and at table (see first paragraph in S1 File and Table A and Table B in S1 File).

### Imputation of missing salt intake or blood pressure data

In case of incomplete data, we contacted authors to obtain the missing data. When authors did not respond to our requests or when this data was not available, missing data was imputed as follows: we estimated the distribution of salt intake over age and gender from earlier published studies when no distribution of salt intake data was available (Poland). When sex-specific salt intake was not reported, we estimated this by applying the sex-specific salt intake ratio from studies that reported sex-specific salt intake to the overall salt intake (Poland). In those countries where age-specific salt intake was available (France, Italy, Netherlands, Sweden and UK), we could not observe a consistent trend of salt intake over age. Therefore, we assumed that salt intake was similar across all ages. For salt intake estimates that did not include an estimate of discretionary salt use (France, Ireland and Sweden), we assumed that 80% of total salt intake would come from the reported salt intake [14] and added an additional 20%.

Systolic blood pressure levels were mostly available by separate age categories. It was assumed that systolic blood pressure increased linearly over age and missing values for each age were predicted by fitting a regression line through the available systolic blood pressure levels over the crude age categories, taking the midpoint of each age category as fitting point. In addition, it was assumed that the blood pressure distribution was similar for the age categories.

### Salt intake scenarios

Current salt intake distribution was categorized into nine different categories (<4 g/d, 4–6 g/d, 6–8 g/d, 8–10 g/d, 10–12 g/d, 12–14 g/d, 14–16 g/d, 16–18 g/d and >18 g/d). Individuals were distributed over these categories assuming that the sex-specific salt intake in the population followed a lognormal distribution (see first paragraph in S1 File).

We modelled the health gain hypothetically obtained when mean population salt intake was reduced by 30% by shifting the whole salt distribution by 30% down, and then re-categorizing the new salt intake distribution into the salt intake categories (see paragraph 1 in S1 File). We also assessed the potential health gain with the population nutrient goal of 5 grams per day. This was modelled by shifting all individuals from their current intake categories towards the intake category of 4–6 grams per day. Individuals who were already in the <4 g/day or in the 4–6 g/day category remained in this category.

### Modelling salt intake to blood pressure and cardiovascular disease

Salt intake categories were further subdivided into salt intake categories of 0.5 g/d. The prevalence of individuals within these subcategories was based again on the assumed log-normal distribution of salt intake in the population.

The dose-response relation between salt intake *(x)* and systolic blood pressure (SBP) *(y)* was derived from a meta-analyse of He and MacGregor [2], and transformed into a continuous, exponential association, resulting in a higher effect of salt reduction in hypertensive subjects as compared with normotensive subjects:

y=c⋅exp(β⋅x)−α (1)

where *α* equals -105, *β* equals 0.03 and *c* is a person specific (random) coefficient (log-normally distributed) (see second paragraph in S1 File).

Eq 1 was used to calculate the SBP distribution for each salt intake subcategory of 0.5 g/d (see third paragraph in S1 File). The mean and standard deviation of log(c) were chosen so that the mean and standard deviation of the resulting SBP distribution for each age and gender were equal to the SBP distribution in the population. As data on the SBP distributions in the population were based on single measurements, not taking short-term variation into account, we did not take the population standard deviation directly from the publications, but adjusted the standard deviation with a factor 0.6 to remove the within-subject variation from the population standard deviation. Relative risks between usual SBP on one hand and IHD and stroke on the other hand were derived from Lewington et al [15]. Using these relative risks, and Eq 1, a relative risk within each salt intake subcategory was calculated for IHD or stroke. Next, the weighted mean RR within each salt intake category was calculated based on all RRs of the subcategories. This modelling step was repeated for each country and resulted in country-specific relative risks (see fourth paragraph in S1 File and Table C and Table D).

### DYNAMO-HIA

Salt intake categories and the relative risks derived from the association between salt intake, systolic blood pressure and cardiovascular were incorporated in the DYNAMO-HIA model (version 1.2) and were used to estimate the effect of salt reduction on the prevalence of cardiovascular diseases (IHD or stroke) and through this on all-cause mortality. More detailed information on the methodology of DYNAMO-HIA is described elsewhere [16,17]. In short, DYNAMO-HIA is a dynamic, Markov-type model that uses actual population data and accounts for changing population compositions, risk factor prevalence and disease burden in several European countries. DYNAMO-HIA requires demographic data and epidemiological information on incidence, prevalence and disease-specific mortality for relevant diseases, by age and sex. As output, the model estimates summary measures of population health. In the model used in the present study, salt intake influences mortality only through its influence on the incidence of IHD and stroke (see fifth paragraph in S1 File).

We compared the current salt intake with the situation in which the total population has a mean salt reduction of 30% or if salt intake was 5 grams per day for all subjects. We assumed that each individual would maintain the same salt intake over the modelling period. Therefore, we specified zero-transition rates between the salt intake categories. We assessed the health impact over a 20-year period for the population aged ≥18 y. We present the results on the prevalence of IHD, stroke and all-cause mortality, life expectancy and disability-adjusted life expectancy.

### Robustness of the model

We used Monte Carlo simulations to estimate uncertainties around the estimates of our model. We used the upper and lower bound of the effects of reduction on blood pressure based on the confidence intervals of He and MacGregor [2], and the upper and lower bound of the association between blood pressure and CVD as presented by Lewington [15]. These intervals were assumed to have a normal probability distribution. The mean and 95% confidence intervals for 100 simulations was reported (see sixth paragraph in S1 File).

In addition, we examined whether our model was robust in case of missing values and of deviations from current assumptions used in estimating the impact of salt reduction towards 5 grams per day. In these analyses, we included countries with least missing values (Italy, Netherlands, Spain and UK), where we had age and sex specific salt intake available and where salt intake estimates were based on 24-hour urine collections. We examined whether the use of age-specific salt intake or blood pressure levels leads to different outcome estimates compared to using average salt intake or blood pressure.

## Results

### Current salt intake and systolic blood pressure levels and its reduction

Estimated salt intake ranged in men from 9.4 g/d in Finland to 13.3 g/d in Poland, respectively, and in women from 7.3 g/d in Finland to 10.0 g/d in Poland (Table 1). The systolic blood pressure was lowest in Sweden in both men and women (128.0 mmHg and 120.7 mmHg respectively) and highest in men in France (138.5 mmHg) and in women in Poland (133.9 mmHg). The projected salt reductions were highest in Poland in both men and women in both scenarios (Table 1), and lowest in Finland.

**Table 1:** Current salt intake and salt intake in the salt reduction scenarios for the nine countries.

Country	Year	Current salt intake (g/d)		30% salt reduction on population level (g/d)		Salt intake of 5 g/d for all individuals	
		Mean (SD)	Mean (SD)	Mean reduction	Mean reduction	Mean reduction	Mean reduction
		Men	Women	Men	Women	Men	Women
Finland	2002 [8]	9.4 (4.0)	7.3 (2.9)	-2.8	-2.2	-4.4	-2.3
France*	2006–2007 [35]	11.0 (2.8)	8.0 (2.0)	-3.3	-2.4	-6.0	-3.0
Ireland*	2008–2010 [36]	10.4 (2.4)	7.6 (1.8)	-3.1	-2.3	-5.4	-2.6
Italy	2008[37]	11.0 (4.0)	8.6 (3.3)	-3.3	-2.6	-6.0	-3.6
Netherlands	2010 [38]	10.9 (3.9)	7.8 (2.7)	-3.4	-2.3	-5.9	-2.8
Poland	2009 [39]	13.3 (4.0)	10.0 (3.1)	-4.0	-3.0	-8.3	-5.0
Spain	2009 [40]	11.5 (4.8)	8.4 (3.9)	-3.4	-2.5	-6.5	-3.4
Sweden*	2010–2011 [41]	11.4 (2.9)	8.7 (2.1)	-3.4	-2.6	-6.4	-3.7
UK	2008 [42]	9.7 (4.1)	7.7 (4.8)	-2.9	-2.3	-4.7	-2.7

* Salt intake is estimated based on salt intake from food records and includes an estimation of discretionary salt use of 20%.

### Prevalence reduction of IHD, stroke and mortality

Table 2 shows the reduction in the prevalence of individuals with stroke and IHD, expected after a 20-year period in the 30% intake reduction scenario as well as in the 5 gram/day scenario, compared with the current salt intake scenario. For a 30% reduction in salt intake the prevalence of stroke would be reduced by 6.4% (N = 8,200; 95% CI 7,600–8,700) in Finland to 13.5% (N = 106,100; 95% CI 102,300–109,900) in Poland. IHD would be reduced by 4.1% (N = 13,700; 95% CI 12,700–14,600) in Finland to 8.9% in Poland (N = 125,100; 95% CI 119,500–130,800).

**Table 2:** Projected disease prevalence and mortality reduction over 20 years for the population aged 18 to 95 years in nine European countries.

		Stroke			Ischemic heart disease			Mortality		
Country		Current	Reduction	%	Current	Reduction	%	Current	Reduction	%
Finland		127,300			329,500			1,096,800		
	30% salt intake reduction		8,200 (7,600–8,700)	6.4		13,700 (12,700–14,600)	4.1		8,500 (8,000–9,000)	0.8
	5 grams per day		12,900 (12,100–13,600)	10.1		21,800 (20,400–23,300)	6.6		13,500 (12,800–14,200)	1.2
France		942,700			1,084,500			11,831,400		
	30% salt intake reduction		101,700 (97,500–105,800)	10.8		84,400 (80,300–88,500)	7.8		66,500 (63,700–69,300)	0.6
	5 grams per day		153,500 (147,300–159,700)	16.3		129,900 (123,600–136,100)	12.0		102,100 (97,800–106,300)	0.9
Ireland		72,900			176,000			641,800		
	30% salt intake reduction		7,200 (6,900–7,500)	9.9		12,000 (11,400–12,600)	6.8		6,200 (5,900–8,600)	1.0
	5 grams per day		10,200 (9,800–10,600)	14.0		17,500 (16,600–18,400)	10.0		9,000 (8,600–9,300)	1.4
Italy		1,395,200			1,956,500			12,776,800		
	30% salt intake reduction		141,400 (135,500–147,300)	10.1		142,900 (135,500–150,00)	7.3		103,000 (98,600–107,500)	0.8
	5 grams per day		222,900 (213,600–232,100)	16.0		228,300 (216,300–240,200)	11.7		163,300 (156,200–170,300)	1.3
Netherlands		303,200			700,000			3,117,100		
	30% salt intake reduction		33,100 (31,600–34,700)	10.9		48,600 (45,700–51,500)	6.9		29,900 (28,400–31,400)	1.0
	5 grams per day		50,000 (47,600–52,300)	16.5		75,600 (71,100–80,100)	10.8		46,100 (43,800–48,400)	1.5
Poland		788,900			1,408,600			8,345,900		
	30% salt intake reduction		106,100 (102,300–109,900)	13.5		125,100 (119,500–130,800)	8.9		109,500 (105,200–113,700)	1.3
	5 grams per day		181,900 (175,500–188,300)	23.1		218,900 (209,000–228,900)	15.5		191,300 (183,900–198,700)	2.3
Spain		782,700			1,023,700			8,585,500		
	30% salt intake reduction		87,800 (84,000–91,600)	11.2		79,600 (75,200–83,900)	7.8		59,100 (56,400–61,700)	0.7
	5 grams per day		143,200 (137,100–149,300)	18.3		131,500 (124,400–138,600)	12.8		97,400 (93,100–101,700)	1.1
Sweden		183,900			414,900			1,897,500		
	30% salt intake reduction		17,800 (16,800–18,800)	9.7		26,500 (24,700–28,400)	6.4		14,400 (13,600–15,300)	0.8
	5 grams per day		28,100 (26,600–29,700)	15.3		42,700 (39,700–45,700)	10.3		23,100 (21,700–24,400)	1.2
UK		1,336,400			2,612,300			12,416,400		
	30% salt intake reduction		133,900 (127,500–140,200)	10.0		175,600 (165,600–185,500)	6.7		98,500 (93,600–103,400)	0.8
	5 grams per day		202,400 (192,900–211,900)	15.1		268,100 (253,000–283,300)	10.3		149,400 (142,000–156,700)	1.2

Numbers reported are the mean (95% CI) for 100 simulations

The reduction of salt intake to 5 grams per day would reduce the prevalence of stroke from 10.1% (N = 12,900; 95% CI 12,100–13,600) in Finland to 23.1% (N = 181,900; 95% CI 175,500–188,300) in Poland, and of IHD by 6.6% (N = 21,800; 95% CI 20,400–23,300) in Finland to 15.5% (N = 218,900; 95% CI 209,000–228,900) in Poland. Table 2 also shows the number of deaths postponed due to salt reduction. Reduction in mortality due a reduction in CVD ranges from almost 102,100 (95% CI 97,800–106,300) (0.9%) in France to 191,300 (95% CI 183,900–198,700) (2.3%) in Poland.

### Life expectancy and disability adjusted life expectancy

Life expectancy increases in all participating countries after reducing salt intake towards 5 g/d compared with the current salt intake. The life expectancy is increased by 0.2 years in 60 year old males from France, Italy, Spain, Sweden and UK and to 0.5 years in Poland (Table 3). In 60-year old women, the life expectancy gained is somewhat lower. The absolute gain is higher in younger individuals than in older individuals. The increase is disability-adjusted life expectancy is somewhat higher compared to life expectancy (Table 3).

**Table 3:** Current life expectancy (LE) and disability-adjusted life expectancy (DALE) and its prolongation (in years and relative change (%)) for a 20-year old and 60-year old individual as a result of reducing dietary salt intake to 5 grams per day in the nine European countries over lifetime.

		20-year old individual				60-year old individual			
		Men		Women		Men		Women	
		Current situation in years	Gain in years (%)	Current situation in years	Gain in years (%)	Current situation in years	Gain in years (%)	Current situation in years	Gain in years (%)
Finland	LE	56.7	0.5 (0.9)	63.4	0.0 (0)	20.9	0.3 (1.4)	25.4	0.0 (0)
	DALE	51.1	0.7 (1.4)	55.8	0.0 (0)	16.9	0.4 (2.4)	20.2	0.0 (0)
France	LE	57.8	0.3 (0.5)	64.7	0.1 (0.2)	21.8	0.2 (0.9)	26.8	0.0 (0)
	DALE	52.0	0.5 (1.0)	56.6	0.2 (0.4)	17.5	0.3 (1.7)	20.9	0.1 (0.5)
Ireland	LE	58.0	0.4 (0.7)	62.3	0.2 (0.3)	20.8	0.3 (1.4)	24.2	0.2 (0.8)
	DALE	52.5	0.6 (1.1)	55.5	0.2 (0.4)	16.9	0.4 (2.4)	19.4	0.2 (1.0)
Italy	LE	59.1	0.4 (0.7)	64.5	0.2 (0.3)	21.8	0.2 (0.9)	26.0	0.2 (0.8)
	DALE	53.5	0.6 (1.1)	56.9	0.4 (0.7)	17.6	0.4 (2.3)	20.6	0.3 (1.5)
Netherlands	LE	58.2	0.4 (0.7)	62.4	0.2 (0.3)	20.7	0.2 (1.0)	24.4	0.2 (0.8)
	DALE	52.5	0.6 (1.1)	54.8	0.2 (0.4)	16.7	0.4 (2.4)	19.1	0.2 (1.0)
Poland	LE	51.8	0.7 (1.4)	60.2	0.4 (0.7)	17.7	0.4 (2.3)	22.8	0.3 (1.3)
	DALE	45.7	0.9 (2.0)	51.6	0.5 (1.0)	13.7	0.5 (3.6)	17.2	0.4 (2.3)
Spain	LE	58.0	0.3 (0.5)	64.4	0.1 (0.2)	21.4	0.2 (0.9)	26.0	0.1 (0.4)
	DALE	52.4	0.6 (1.1)	57.0	0.3 (0.5)	17.2	0.4 (2.3)	20.5	0.2 (1.0)
Sweden	LE	59.3	0.3 (0.5)	63.4	0.1 (0.2)	21.8	0.2 (0.9)	25.1	0.1 (0.4)
	DALE	53.3	0.6 (1.1)	55.8	0.2 (0.4)	17.6	0.4 (2.3)	19.8	0.2 (1.0)
UK	LE	57.9	0.3 (0.5)	62.0	0.2 (0.3)	21.1	0.2 (0.9)	24.2	0.1 (0.4)
	DALE	51.9	0.5 (1.0)	54.4	0.3 (0.6)	16.9	0.3 (1.8)	19.0	0.2 (1.0)

### Robustness of model in case of missing values and/or assumptions made (sensitivity analyses)

**Fig 1:**
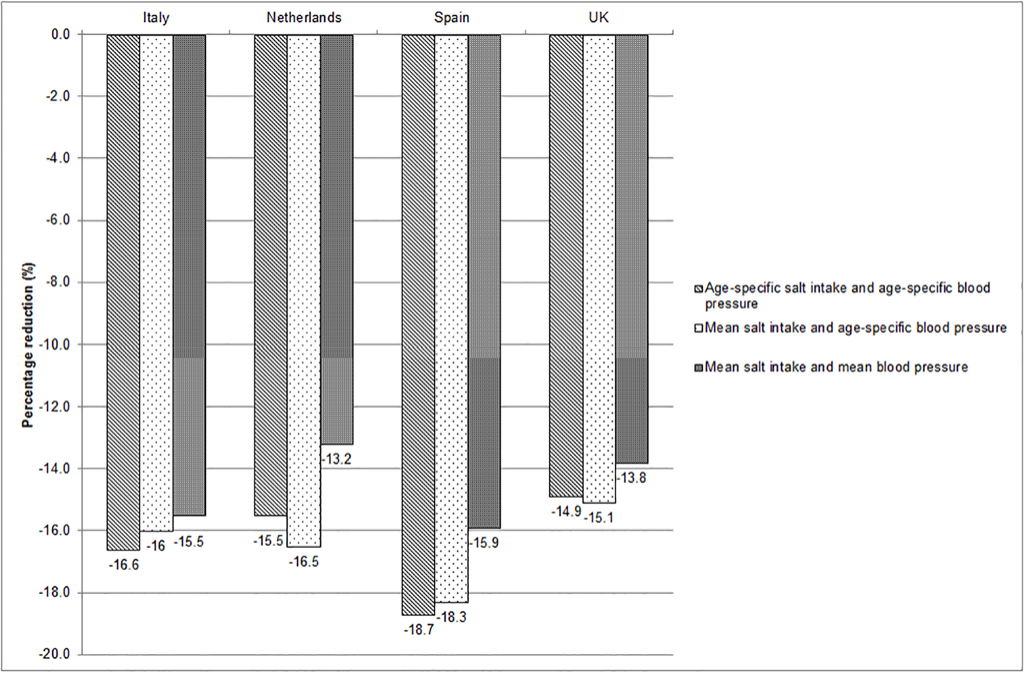
Percentage reduction in prevalence of stroke due to modification of input parameters salt intake and systolic blood pressure (for Italy, Netherlands, Spain and UK).

We observed a stronger reduction in the prevalence of stroke in the Netherlands and UK when we included average salt intake in our model as compared with age-specific salt intake (Fig 1), especially in the UK. This is because the average salt intake in the UK population was higher than the salt intake in the UK elderly, particularly the part of the population that will benefit most from salt reduction. If average blood pressure levels were included in the DYNAMO-HIA model the health impact estimates were lower as compared with age-specific salt intake and age-specific blood pressure levels in all countries.

## Discussion

Using the dynamic population health modelling tool DYNAMO-HIA, we have been able to quantify the potential impact of a 30% salt reduction in the population and the effect of a reduction to 5 grams per day for all individuals on cardiovascular diseases and mortality in nine European countries. It is the first study that is able to compare the variation in cardiovascular diseases and mortality that could be averted due to salt reduction in nine European countries. A 30% salt reduction will already reduce the prevalence of stroke and IHD and will reduce the number of deaths in all nine European countries. Salt reduction to 5 grams per day will lead to further reductions in cardiovascular diseases and mortality.

The cardiovascular disease prevalence and averted cases of cardiovascular disease by salt reduction varied across the European countries; Poland would have more than twice as big a reduction in CVD than Finland, where salt intake has already been lowered substantially over the last few decades. In general, high salt intake is the main determinant of stronger reductions in cardiovascular diseases. Moreover, we observed that health benefits of salt reduction are mediated by systolic blood pressure. Countries with raised current blood pressure levels (e.g. Ireland) have more beneficial effects of salt reduction as compared with countries with lower current blood pressure (e.g. Netherlands) although salt intake is comparable between the Netherlands and Ireland. The calculations showed a relatively small reduction in all-cause mortality because the DYNAMO-HIA model assumes no direct association between salt intake and mortality, but only an indirect relation through the incidence of diseases. However, the all-cause reduction in mortality will be mainly due to the reduction in cardiovascular disease mortality. The increase in life expectancy and disability-adjusted life is relatively higher in older individuals compared with younger individuals, because this intervention is more likely to postpone mortality in older adults than in younger adults.

This study shows that salt reduction below the population nutrient goal of 5 grams per day as recommended by WHO will have substantial benefits for public health across the WHO European Region. Although the salt intake levels vary across the countries, all intakes exceed the WHO population nutrient goal of 5 grams per day. Substantial reductions are needed to reach this goal (on average 47%), and the intermediate reduction of 30% is considered a more realistic target to be achieved in 2025 [18]. Generally, processed foods are the main contributor to salt intake in West-European countries and major salt reduction can only be achieved if sodium levels of processed foods are significantly reduced.

In several European countries, voluntary sodium reductions have been implemented. In the UK, for example, the salt reduction program has been underway since 2003. Over this period, sodium levels in several processed foods decreased by 30 to 40%, and consumer awareness of high-salt products and use of discretionary salt increased [19,20]. The concurrent salt reduction in the population was 15% (from 9.5 g/d in 2001 to 8.1 g/d in 2011) [9], but the actual impact on blood pressure and cardiovascular disease is yet unknown. In Finland and the UK, the population-wide salt reduction co-incided with simultaneous reduction in blood pressure, but causality cannot be conclusively inferred from such an ecological association [21,22]. Only a restricted number of population-based interventions studies have been carried out that aimed to reduce the populations’ salt intake and evaluate its effect on blood pressure levels. Some studies show that reduction in salt intake could not be achieved or maintained and that therefore no change in blood pressure levels could be observed [23,24]. Another study that did successfully decrease salt intake demonstrated a reduction in blood pressure in the population [25] was critized due to the inclusion of many hypertensive subjects which meant that the results could not be extrapolated to the general population [26]. In the present study, we adapted the dose-response association between salt intake and blood pressure from a meta-analysis of He and MacGregor, which included intervention studies with duration of 4 weeks or more [2]. However, the median duration of those interventions was 4 weeks and it can be questioned whether the effect observed in these studies will last over our 20 year simulation period.

Several modelling studies have concluded that sodium reduction in processed foods (mandatory or voluntary) is a very cost-effective measure, as the benefits in terms of cost reduction would significantly outweigh the costs of the intervention [10,12,27–29]. Cobiac *et al* argues that programmes that stimulate the food industry to reduce the sodium levels in processed foods voluntarily may be recommended to improve public health, but it is anticipated that regulatory actions will be needed to achieve the maximal potential effect on public health [12], simply because this will result in lower salt intake levels.

This health modelling tool can be used to compare the health impacts of salt reduction scenarios between European countries and thus explore the variation in potential health impact. We took into account the variation in salt intake and blood pressure distributions in the nine countries and included country-specific demographic and cardiovascular disease prevalence data in the DYNAMO-HIA model. Moreover, we modelled an increased salt sensitivity for hypertensive individuals because of a convincing stronger dose-response association for hypertensive individuals compared with normotensive individuals reported in literature [2]. However, modelling is always a simplification of reality and is limited by the model input parameters. We had to impute data if this was not available (estimation of discretionary salt, sex-specific or distribution of salt intake). Moreover, we imputed age-specific systolic blood pressure levels based on blood pressure levels that were presented according to crude age categories. We tested the robustness of our model to explore the sensitivity on the outcomes of the model when we modified the input parameter salt intake and blood pressure on the outcomes of the model. We observed that age-specific salt intake yielded quite similar results compared to mean salt intake, unless salt intake in older adults is considerable lower than the average salt intake, as observed in the UK. In contrast, one mean blood pressure estimate will substantially reduce the modelled health benefits of salt reduction, likely due to the increase in blood pressure at older age and the subsequent increase in incidence and prevalence of stroke and IHD [15]. These robustness analyses show that our current strategy to impute missing data still provided acceptable estimate and that population mean salt intake can be used as a proxy for age-specific salt intake. Unfortunately, our model did not include other diseases that have been associated with high salt intake, such as congestive heart failure [30–32]. In addition, we were not able to incorporate other effects related to salt reduction, such as kidney diseases, osteoporosis or gastric cancer [33,34]. Therefore, the projected effects are likely to be an underestimation of the overall effects that salt reduction can achieve.

In conclusion, this study shows that reducing salt intake to maximally 5 grams per day will substantially reduce the prevalence of cardiovascular diseases across nine European countries. Salt reduction will largely contribute to the reduction in burden of NCDs. Even a 30% mean salt reduction is beneficial for public health, and may be a more realistic target to achieve by 2025.

## Supporting Information

S1 File Paragraph 1 to 6 in S1 File include a more detailed description of the modelling approach. It also includes information on the salt intake distribution (Table A and B in S1 File) and the combined relative risks between salt intake and stroke and IHD (Table C and D in S1 File).
(DOCX)
