# Supplementary material for: Health Gain by Salt Reduction in Europe: A Modelling Study
Source: PLoS One. 2015 Mar 31;10(3):e0118873. doi: 10.1371/journal.pone.0118873 (PMC4380413; doi:10.1371/journal.pone.0118873)
Supplement: S1 File — It also includes information on the salt intake distribution (Table A and B in S1 File) and the combined relative risks between salt intake and stroke and IHD (Table C and D in S1 File). (DOCX) [file pone.0118873.s001.docx]

**Supporting information to ‘HEALTH GAIN BY SALT INTAKE REDUCTION IN EUROPE: A MODELLING STUDY’**

**1. Salt intake distribution**

The prevalence of salt intake over the salt intake categories in the current situation, 30% salt reduction and in 5 grams per day for men and women are presented in Table A and B

*Table A. Prevalence of salt intake over the salt intake categories in the current situation, 30% salt intake reduction and in 5 grams per day for men*

|  |  | **Men** |  |  |  |  |  |  |  |  |
| --- | --- | --- | --- | --- | --- | --- | --- | --- | --- | --- |
|  |  | **<4 g/d** | **4-6 g/d** | **6-8 g/d** | **8-10 g/d** | **10-12 g/d** | **12-14 g/d** | **14-16 g/d** | **16-18 g/d** | **>18 g/d** |
| Finland | Current | 0.1 | 3.4 | 18.1 | 28.8 | 24.2 | 14.2 | 6.7 | 2.8 | 1.8 |
|  | 30% reduction | 2.4 | 27.4 | 39.3 | 21.2 | 7.2 | 2.0 | 0.5 | 0.1 | 0.0 |
|  | 5 g/d | 0.1 | 99.9 | 0.0 | 0.0 | 0.0 | 0.0 | 0.0 | 0.0 | 0.0 |
| France | Current | 0.0 | 1.0 | 11.2 | 27.1 | 28.3 | 18.2 | 8.7 | 3.5 | 1.9 |
|  | 30% reduction | 0.6 | 18.5 | 41.8 | 27.0 | 9.2 | 2.3 | 0.5 | 0.1 | 0.0 |
|  | 5 g/d | 0.0 | 100 | 0.0 | 0.0 | 0.0 | 0.0 | 0.0 | 0.0 | 0.0 |
| Ireland | Current | 0.0 | 0.81 | 12.9 | 33.1 | 30.5 | 15.3 | 5.4 | 1.5 | 0.5 |
|  | 30% reduction | 0.5 | 21.8 | 48.4 | 23.4 | 5.1 | 0.8 | 0.1 | 0.0 | 0.0 |
|  | 5 g/d | 0.0 | 100 | 0.0 | 0.0 | 0.0 | 0.0 | 0.0 | 0.0 | 0.0 |
| Italy | Current | 0.3 | 5.5 | 17.0 | 22.9 | 20.3 | 14.3 | 8.8 | 5.0 | 5.8 |
|  | 30% reduction | 4.5 | 25.0 | 31.7 | 21.0 | 10.4 | 4.5 | 1.8 | 0.7 | 0.5 |
|  | 5 g/d | 0.3 | 99.7 | 0.0 | 0.0 | 0.0 | 0.0 | 0.0 | 0.0 | 0.0 |
| Netherlands | Current | 0.5 | 6.9 | 19.1 | 23.9 | 19.8 | 13.2 | 7.8 | 4.3 | 4.6 |
|  | 30% reduction | 5.4 | 27.2 | 31.9 | 19.9 | 9.4 | 3.9 | 1.5 | 0.6 | 0.4 |
|  | 5 g/d | 0.5 | 99.5 | 0.0 | 0.0 | 0.0 | 0.0 | 0.0 | 0.0 | 0.0 |
| Poland | Current | 0.0 | 0.3 | 4.3 | 14.2 | 22.0 | 21.7 | 16.2 | 10.1 | 11.2 |
|  | 30% reduction | 0.2 | 7.1 | 26.1 | 31.0 | 20.3 | 9.6 | 3.8 | 1.4 | 0.7 |
|  | 5 g/d | 0.0 | 100 | 0.0 | 0.0 | 0.0 | 0.0 | 0.0 | 0.0 | 0.0 |
| Spain | Current | 0.7 | 7.0 | 16.4 | 20.1 | 18.0 | 13.5 | 9.2 | 5.9 | 9.2 |
|  | 30% reduction | 5.9 | 23.3 | 27.6 | 19.9 | 11.5 | 6.0 | 3.0 | 1.4 | 1.4 |
|  | 5 g/d | 0.7 | 99.3 | 0.0 | 0.0 | 0.0 | 0.0 | 0.0 | 0.0 | 0.0 |
| Sweden | Current | 0.0 | 0.7 | 9.2 | 24.9 | 28.5 | 19.8 | 10.1 | 4.3 | 2.5 |
|  | 30% reduction | 0.4 | 14.7 | 39.6 | 29.7 | 11.5 | 3.2 | 0.7 | 0.2 | 0.0 |
|  | 5 g/d | 0.0 | 100 | 0.0 | 0.0 | 0.0 | 0.0 | 0.0 | 0.0 | 0.0 |
| UK | Current | 2.5 | 14.1 | 22.8 | 21.4 | 15.6 | 9.9 | 5.9 | 3.4 | 4.3 |
|  | 30% reduction | 14.0 | 32.3 | 26.7 | 14.7 | 6.9 | 3.1 | 1.3 | 0.6 | 0.5 |
|  | 5 g/d | 2.5 | 97.5 | 0.0 | 0.0 | 0.0 | 0.0 | 0.0 | 0.0 | 0.0 |

*Table B. Prevalence of salt intake over the salt intake categories in the current situation, 30% salt intake reduction and in 5 grams per day for women*

|  |  | **Women** |  |  |  |  |  |  |  |  |
| --- | --- | --- | --- | --- | --- | --- | --- | --- | --- | --- |
|  |  | **<4 g/d** | **4-6 g/d** | **6-8 g/d** | **8-10 g/d** | **10-12 g/d** | **12-14 g/d** | **14-16 g/d** | **16-18 g/d** | **>18 g/d** |
| Finland | Current | 0.9 | 21.7 | 42.9 | 24.7 | 7.7 | 1.8 | 0.3 | 0.1 | 0.0 |
|  | 30% reduction | 16.5 | 57.6 | 22.3 | 3.3 | 0.3 | 0.0 | 0.0 | 0.0 | 0.0 |
|  | 5 g/d | 0.9 | 99.1 | 0.0 | 0.0 | 0.0 | 0.0 | 0.0 | 0.0 | 0.0 |
| France | Current | 0.4 | 14.7 | 39.6 | 29.7 | 11.5 | 3.2 | 0.7 | 0.2 | 0.0 |
|  | 30% reduction | 11.2 | 54.6 | 28.2 | 5.3 | 0.6 | 0.1 | 0.0 | 0.0 | 0.0 |
|  | 5 g/d | 0.4 | 99.6 | 0.0 | 0.0 | 0.0 | 0.0 | 0.0 | 0.0 | 0.0 |
| Ireland | Current | 0.4 | 17.9 | 45.2 | 27.1 | 7.7 | 1.5 | 0.2 | 0.0 | 0.0 |
|  | 30% reduction | 13.5 | 61.0 | 22.7 | 2.6 | 0.2 | 0.01 | 0.0 | 0.0 | 0.0 |
|  | 5 g/d | 0.4 | 99.6 | 0.0 | 0.0 | 0.0 | 0.0 | 0.0 | 0.0 | 0.0 |
| Italy | Current | 3.0 | 18.8 | 28.1 | 22.7 | 13.7 | 7.2 | 3.5 | 1.6 | 1.4 |
|  | 30% reduction | 17.6 | 39.0 | 26.1 | 11.2 | 4.0 | 1.4 | 0.5 | 0.2 | 0.1 |
|  | 5 g/d | 3.0 | 97.0 | 0.0 | 0.0 | 0.0 | 0.0 | 0.0 | 0.0 | 0.0 |
| Netherlands | Current | 3.6 | 23.5 | 32.2 | 22.1 | 11.0 | 4.5 | 1.9 | 0.7 | 0.4 |
|  | 30% reduction | 21.2 | 45.4 | 23.7 | 7.3 | 1.8 | 0.4 | 0.1 | 0.0 | 0.0 |
|  | 5 g/d | 3.6 | 96.4 | 0.0 | 0.0 | 0.0 | 0.0 | 0.0 | 0.0 | 0.0 |
| Poland | Current | 0.0 | 3.3 | 19.9 | 31.8 | 24.6 | 12.6 | 5.1 | 1.8 | 0.9 |
|  | 30% reduction | 2.2 | 30.3 | 41.8 | 19.2 | 5.2 | 1.1 | 0.2 | 0.0 | 0.0 |
|  | 5 g/d | 0.0 | 100 | 0.0 | 0.0 | 0.0 | 0.0 | 0.0 | 0.0 | 0.0 |
| Spain | Current | 7.2 | 22.2 | 25.1 | 18.7 | 11.7 | 6.7 | 3.7 | 2.0 | 2.5 |
|  | 30% reduction | 25.9 | 34.9 | 21.5 | 10.1 | 4.3 | 1.8 | 0.8 | 0.3 | 0.3 |
|  | 5 g/d | 7.2 | 92.8 | 0.0 | 0.0 | 0.0 | 0.0 | 0.0 | 0.0 | 0.0 |
| Sweden | Current | 0.1 | 6.4 | 33.1 | 36.4 | 17.3 | 5.2 | 1.2 | 0.2 | 0.1 |
|  | 30% reduction | 4.3 | 47.7 | 38.3 | 8.5 | 1.0 | 0.1 | 0.0 | 0.0 | 0.0 |
|  | 5 g/d | 0.1 | 99.9 | 0.0 | 0.0 | 0.0 | 0.0 | 0.0 | 0.0 | 0.0 |
| UK | Current | 19.8 | 24.7 | 19.8 | 13.3 | 8.4 | 5.1 | 3.2 | 2.0 | 3.7 |
|  | 30% reduction | 40.7 | 27.6 | 15.4 | 7.8 | 4.0 | 2.0 | 1.1 | 0.6 | 0.8 |
|  | 5 g/d | 19.8 | 80.2 | 0.0 | 0.0 | 0.0 | 0.0 | 0.0 | 0.0 | 0.0 |

**2. Dose-response association between salt intake and blood pressure**

We reformulated the findings of He and MacGregor [[1](#_ENREF_1)] in which we assumed that the change in SBP per additional amount of salt in the diet is linearly related to the SBP levels:

$\frac{dSBP}{dSI}=\beta(\alpha+SBP)$

where *SI* is salt intake (g/d), SBP is SBP level (mmHg), and *α* and *β* are regression coefficients of the dose-response curve. Solving equation (5) results in the following formula of the SBP level as a function of salt intake:

$$SBP={ce}^{\beta SI}-\alpha$$

with *c* a stochastic parameter depending on age and sex. According to He and MacGregor, the SBP level of 127 mmHg (normal SBP) corresponds to a $\frac{dSBP}{dSI}$ of 0.6 mmHg/g salt and a SBP level of 149 mmHg (hypertension) corresponds to a $\frac{dSBP}{dSI}$ of 1.2 mmHg/g salt. Using these two findings as conditions, the regression coefficients *α* and *β* are estimated; $\alpha=105$, and$\beta=0.028$. We assumed that blood pressure levels of ≥ 160 mmHg lead to a maximal reduction of 1.5 mmHg per gram salt intake reduction.

The variation of the SBP level in the population is larger than the variation resulting from the variation in salt intake. Therefore, we assumed that *c* is a stochastic parameter that is lognormally distributed. The mean and standard deviation of log(C) was estimated by equating the mean and variance of the SBP calculated from salt intake using the formulae above to the observed mean and variance in the population.

**3. Blood pressure distribution within each salt intake category**

Salt intake categories were further subdivided into salt intake categories of 0.5 g/d. In each salt intake category, 100 blood pressure values were calculated representing the current blood pressure distribution.

**4. Relative risks for disease incidence**

For each of the 100 blood pressures representing the blood pressure in a salt category, a single relative risk was calculated using the dose-response relation between blood pressure and IHD or stroke. We used the relative risks for incidence of IHD and stroke that were presented by Lewington [[2](#_ENREF_2)]. For each broader salt category, the average of these relative risks was taken. This average was weighted for the blood pressure distribution over these subcategories. All relative risks were divided by the relative risk for the salt category 4-6 g/d, in order to make the latter the reference category.

Table C and table D give the resulting relative risks at the ages of 40, 60 and 80 years for stroke and IHD.

*Table C. Combined relative risks between salt intake and stroke for a selection of salt intake categories and for selected ages*

|  |  | **Men** |  |  |  | **Women** |  |  |  |
| --- | --- | --- | --- | --- | --- | --- | --- | --- | --- |
|  | **Age^1^** | **<4 g/d^2^** | **4-6 g/d** | **8-10 g/d** | **14-16 g/d** | **<4 g/d** | **4-6 g/d** | **8-10 g/d** | **14-16 g/d** |
| Finland | 40 y | 0.93 | 1 | 1.18 | 1.60 | 0.99 | 1 | 1.00 | 1.00 |
|  | 60 y | 0.92 | 1 | 1.20 | 1.67 | 0.99 | 1 | 1.00 | 1.00 |
|  | 80 y | 0.96 | 1 | 1.10 | 1.29 | 0.99 | 1 | 1.00 | 1.00 |
| France | 40 y | 0.93 | 1 | 1.16 | 1.54 | 0.94 | 1 | 1.16 | 1.52 |
|  | 60 y | 0.92 | 1 | 1.20 | 1.67 | 0.93 | 1 | 1.19 | 1.64 |
|  | 80 y | 0.96 | 1 | 1.09 | 1.28 | 0.96 | 1 | 1.09 | 1.27 |
| Ireland | 40 y | 0.94 | 1 | 1.15 | 1.51 | 0.94 | 1 | 1.15 | 1.50 |
|  | 60 y | 0.92 | 1 | 1.20 | 1.68 | 0.92 | 1 | 1.22 | 1.73 |
|  | 80 y | 0.96 | 1 | 1.10 | 1.30 | 0.96 | 1 | 1.10 | 1.30 |
| Italy | 40 y | 0.94 | 1 | 1.16 | 1.53 | 0.94 | 1 | 1.16 | 1.50 |
|  | 60 y | 0.92 | 1 | 1.20 | 1.65 | 0.92 | 1 | 1.21 | 1.68 |
|  | 80 y | 0.96 | 1 | 1.09 | 1.27 | 0.96 | 1 | 1.10 | 1.29 |
| Netherlands | 40 y | 0.94 | 1 | 1.15 | 1.50 | 0.95 | 1 | 1.14 | 1.46 |
|  | 60 y | 0.93 | 1 | 1.20 | 1.64 | 0.93 | 1 | 1.19 | 1.61 |
|  | 80 y | 0.96 | 1 | 1.09 | 1.27 | 0.97 | 1 | 1.08 | 1.25 |
| Poland | 40 y | 0.94 | 1 | 1.15 | 1.49 | 0.94 | 1 | 1.15 | 1.48 |
|  | 60 y | 0.92 | 1 | 1.19 | 1.61 | 0.91 | 1 | 1.22 | 1.74 |
|  | 80 y | 0.97 | 1 | 1.08 | 1.24 | 0.96 | 1 | 1.11 | 1.32 |
| Spain | 40 y | 0.94 | 1 | 1.16 | 1.51 | 0.94 | 1 | 1.15 | 1.47 |
|  | 60 y | 0.92 | 1 | 1.22 | 1.69 | 0.92 | 1 | 1.21 | 1.67 |
|  | 80 y | 0.96 | 1 | 1.09 | 1.27 | 0.96 | 1 | 1.10 | 1.30 |
| Sweden | 40 y | 0.94 | 1 | 1.14 | 1.48 | 0.94 | 1 | 1.15 | 1.48 |
|  | 60 y | 0.93 | 1 | 1.17 | 1.54 | 0.93 | 1 | 1.17 | 1.58 |
|  | 80 y | 0.97 | 1 | 1.07 | 1.21 | 0.97 | 1 | 1.08 | 1.23 |
| UK | 40 y | 0.93 | 1 | 1.20 | 1.64 | 0.93 | 1 | 1.17 | 1.53 |
|  | 60 y | 0.92 | 1 | 1.21 | 1.66 | 0.91 | 1 | 1.21 | 1.68 |
|  | 80 y | 0.96 | 1 | 1.09 | 1.26 | 0.95 | 1 | 1.09 | 1.28 |

^1^ DYNAMO-HIA is divided into one-year age categories. Numbers presented only for selected ages

^2^ Salt intake is divided into nine salt intake categories. Numbers presented only for selected categories

*Table D. Combined relative risks between salt intake and IHD for a selection of salt intake categories and for selected ages*

|  |  | **Men** |  |  |  | **Women** |  |  |  |
| --- | --- | --- | --- | --- | --- | --- | --- | --- | --- |
|  | **Age^1^** | **<4 g/d^2^** | **4-6 g/d** | **8-10 g/d** | **14-16 g/d** | **<4 g/d** | **4-6 g/d** | **8-10 g/d** | **14-16 g/d** |
| Finland | 40 y | 0.95 | 1 | 1.12 | 1.35 | 0.99 | 1 | 1.00 | 1.00 |
|  | 60 y | 0.95 | 1 | 1.12 | 1.38 | 0.99 | 1 | 1.00 | 1.00 |
|  | 80 y | 0.97 | 1 | 1.08 | 1.24 | 0.99 | 1 | 1.00 | 1.00 |
| France | 40 y | 0.96 | 1 | 1.10 | 1.31 | 0.97 | 1 | 1.07 | 1.23 |
|  | 60 y | 0.95 | 1 | 1.12 | 1.38 | 0.96 | 1 | 1.11 | 1.35 |
|  | 80 y | 0.96 | 1 | 1.09 | 1.24 | 0.97 | 1 | 1.08 | 1.23 |
| Ireland | 40 y | 0.96 | 1 | 1.09 | 1.30 | 0.97 | 1 | 1.08 | 1.26 |
|  | 60 y | 0.95 | 1 | 1.13 | 1.39 | 0.95 | 1 | 1.13 | 1.40 |
|  | 80 y | 0.96 | 1 | 1.08 | 1.25 | 0.97 | 1 | 1.08 | 1.25 |
| Italy | 40 y | 0.96 | 1 | 1.09 | 1.30 | 0.97 | 1 | 1.08 | 1.24 |
|  | 60 y | 0.95 | 1 | 1.12 | 1.37 | 0.95 | 1 | 1.12 | 1.38 |
|  | 80 y | 0.97 | 1 | 1.08 | 1.23 | 0.97 | 1 | 1.08 | 1.25 |
| Netherlands | 40 y | 0.96 | 1 | 1.10 | 1.30 | 0.97 | 1 | 1.07 | 1.22 |
|  | 60 y | 0.95 | 1 | 1.12 | 1.36 | 0.96 | 1 | 1.11 | 1.33 |
|  | 80 y | 0.97 | 1 | 1.08 | 1.23 | 0.97 | 1 | 1.07 | 1.21 |
| Poland | 40 y | 0.96 | 1 | 1.09 | 1.29 | 0.96 | 1 | 1.09 | 1.27 |
|  | 60 y | 0.95 | 1 | 1.11 | 1.33 | 0.94 | 1 | 1.14 | 1.42 |
|  | 80 y | 0.97 | 1 | 1.07 | 1.20 | 0.96 | 1 | 1.10 | 1.27 |
| Spain | 40 y | 0.96 | 1 | 1.09 | 1.28 | 0.97 | 1 | 1.08 | 1.23 |
|  | 60 y | 0.95 | 1 | 1.12 | 1.38 | 0.95 | 1 | 1.13 | 1.38 |
|  | 80 y | 0.97 | 1 | 1.08 | 1.23 | 0.96 | 1 | 1.09 | 1.25 |
| Sweden | 40 y | 0.96 | 1 | 1.09 | 1.27 | 0.97 | 1 | 1.07 | 1.22 |
|  | 60 y | 0.96 | 1 | 1.09 | 1.29 | 0.96 | 1 | 1.10 | 1.31 |
|  | 80 y | 0.97 | 1 | 1.06 | 1.17 | 0.97 | 1 | 1.06 | 1.19 |
| UK | 40 y | 0.95 | 1 | 1.12 | 1.37 | 0.96 | 1 | 1.10 | 1.29 |
|  | 60 y | 0.95 | 1 | 1.12 | 1.37 | 0.94 | 1 | 1.12 | 1.37 |
|  | 80 y | 0.97 | 1 | 1.08 | 1.22 | 0.96 | 1 | 1.08 | 1.23 |

^1^ DYNAMO-HIA is divided into one-year age categories. Numbers presented only for selected ages

^2^ Salt intake is divided into nine salt intake categories. Numbers presented only for selected categories

**5. DYNAMO-HIA model in more detail**

The DYNAMIC MODEL for Health Impact Assessment (DYNAMO-HIA) is a Markov model combining micro-simulation of the exposure variable with macro-simulation of the disease and survival. For more details, see Boshuizen et al, who published a detailed description of the model [[3](#_ENREF_3)]. In the present study, we used DYNAMO-HIA version 1.2 (available at the website www.dynamo-hia.eu). We simulated the effect of the population aged 18 years and older and our simulated population size was 100 subjects in each salt intake category. We did not include any newborns in our simulation. The transition rates between the risk factor categories were assumed to be zero. We only included chronic diseases in our model that were causally related to systolic blood pressure: IHD and stroke.

*Incidence, prevalence and excess mortality data included in the DYNAMO-HIA model*

A detailed description of the data collection of the prevalence, incidence and excess mortality can be found at the website of DYNAMO-HIA ([www.dynamo-hia.eu](http://www.dynamo-hia.eu); Report on the data collection for cardiovascular disease and diabetes and related relative risks, 2010).

In short, generally data collection of IHD differs widely and different definitions and procedures are in use to select events (f.e. population based registries, or GP networks). GP networks are considered to provide the best data for estimating prevalence and incidence, excess mortality and 28-day case fatality, and are available in UK (UK GPRD) and the Netherlands.

In order to obtain comparable IHD data across countries, IHD mortality and incidence rates were extracted for each country (based on the available registries) and a ratio was calculated in relation to the UK. This ratio was then applied to the UK GPRD IHD incidence data for all other countries (except the Netherlands). All incidence data was subject to incidence-prevalence and mortality modelling in order to obtain consistent estimates for prevalence and excess mortality (using RR from UK GPRD registry). In addition, 28 day case fatality was obtained from GPRD as well.

Stroke incidence and prevalence was obtained from GP registries from the UK and the Netherlands. A review of available stroke incidence and prevalence data in Europe was available [[4](#_ENREF_4)] and provided best estimates in the other countries.

**6. Uncertainty analyses**

Monte Carlo simulations were used to estimate the uncertainties around the model estimates. We used the lower and upper estimates of the effects of reduction on blood pressure based on the confidence intervals of He and MacGregor [[1](#_ENREF_1)], and the lower and upper estimates of the association between blood pressure and IHD and stroke based on the confidence intervals of the log(RR) of Lewington [[2](#_ENREF_2)]. The intervals of both associations were assumed to have a normal probability distribution. The mean and 95% confidence interval of 100 simulations are presented.

**References**

1. He FJ, MacGregor GA (2004) Effect of longer-term modest salt reduction on blood pressure. Cochrane Database Syst Rev: CD004937.

2. Lewington S, Clarke R, Qizilbash N, Peto R, Collins R (2002) Age-specific relevance of usual blood pressure to vascular mortality: a meta-analysis of individual data for one million adults in 61 prospective studies. Lancet 360: 1903-1913

3. Boshuizen HC, Lhachimi SK, van Baal PH, Hoogenveen RT, Smit HA, Mackenbach JP, et al. (2012) The DYNAMO-HIA Model: An Efficient Implementation of a Risk Factor/Chronic Disease Markov Model for Use in Health Impact Assessment (HIA). Demography 49: 1259-1583.

4. Truelsen T, Piechowski-Jozwiak B, Bonita R, Mathers C, Bogousslavsky J, Boysen G (2006) Stroke incidence and prevalence in Europe: a review of available data*.* Eur J Neurol 13: 581-98.
